# Klotho G‐395A Polymorphism as a Risk Factor for Atrial Fibrillation in Patients on Maintenance Hemodialysis

**DOI:** 10.1155/aiu/9179415

**Published:** 2026-09-06

**Authors:** Ying Zhang, Xiangxiang Wang, XiaoXuan Su, Zhixiang Bian, Shunjie Chen

**Affiliations:** ^1^ Nephrology Department, Shanghai Fourth People’s Hospital, School of Medicine, Tongji University, Shanghai, China, tongji.edu.cn

**Keywords:** atrial fibrillation, intact fibroblast growth factor 23, klotho G-395A, left atrial diameter, maintenance hemodialysis, soluble klotho protein

## Abstract

**Objective:**

To explore the relationship between atrial fibrillation and the Klotho G‐395A polymorphism in patients on maintenance hemodialysis (MHD).

**Methods:**

The study included 120 MHD patients (persistent atrial fibrillation, *n* = 30; paroxysmal atrial fibrillation, *n* = 30; normal sinus rhythm, *n* = 60) and 120 control individuals. Klotho G‐395A mutations were detected by the fluorescence quantitative polymerase chain reaction. ELISA was used to detect serum soluble Klotho (sKl) and intact fibroblast growth factor 23 (iFGF23) levels, and left atrium diameters were measured by echocardiography. Correlation analysis between Klotho G‐395A genotypes, clinical parameters, and biochemical indexes was performed, along with logistic regression analysis to determine the risk factors for atrial fibrillation.

**Results:**

Three genotypes of Klotho G‐395A, namely, GG, GA, and AA, were detected. The frequencies of the GA/AA genotypes and A allelic genes were significantly higher in the MHD patients than in the healthy controls, and these genotypes were associated with both atrial fibrillation and persistent atrial fibrillation. Furthermore, serum sKl, serum iFGF23, and left artery diameter were significantly associated with atrial fibrillation and also showed significant differences between the persistent and paroxysmal atrial fibrillation groups. The serum sKl level was negatively correlated with serum FGF23 level and the left atrium diameter, while the serum FGF23 level was positively correlated with the left atrium diameter. The incidence of atrial fibrillation and left atrium diameter was significantly higher in those with the GA/AA genotype than in those with the GG genotype, while the serum sKl levels were significantly lower. The GA/AA genotype was associated with a 5.444 times higher likelihood of atrial fibrillation than the GG genotype.

**Conclusions:**

The A allele of Klotho G‐395A may be a risk marker of atrial fibrillation in MHD patients. Moreover, sKl deficiency may contribute to the early development of atrial fibrillation in MHD patients.

## 1. Introduction

Cardiovascular events are the most common cause of death in patients on maintenance hemodialysis (MHD), accounting for 39%–40% of all deaths in MHD patients [1–4]. Atrial fibrillation is a common type of arrhythmia in MHD patients that greatly increases the risk of death in MHD patients complicated with cardiovascular disease [5, 6]. In recent years, an increasing number of studies have been conducted on atrial fibrillation in MHD patients. Traditionally, the risk factors for atrial fibrillation include hypertension and volume overload [7, 8]. However, the occurrence of atrial fibrillation cannot be completely avoided even after actively controlling for these factors in the clinical setting. Therefore, the focus has shifted to genetic risk factors that can help identify MHD patients who are at risk.

As it is known that the incidence of atrial fibrillation increases with age, the antiaging gene Klotho has been receiving increasing attention in the cardiovascular field. This gene encodes the Klotho protein and is mainly expressed in tissues or organs such as the kidney and thyroid glands [9]. The Klotho protein is divided into two types—membrane Klotho protein (mKl) and soluble Klotho protein (sKl). The sKl protein is formed by the cleavage of the mKl protein, which is expressed on the surface of cell membranes, which can increase the affinity of intact fibroblast growth factor 23 (iFGF23) and its receptor [10]. sKl acts as a phosphate regulatory hormone that promotes the excretion of phosphate in renal tubules and inhibits the production of 1,25‐dihydroxyvitamin D3 [11]. In addition, sKl can also exert antifibrosis, antioxidative stress, antiapoptosis, and other beneficial effects via various mechanisms [12]. Studies have found that the occurrence of atrial fibrillation may be related to the decrease in the levels of the sKl protein or increase in the levels of iFGF23 [13, 14]. Moreover, abnormal expression of the Klotho gene plays a very important role in the occurrence and development of kidney diseases, such as IgA nephropathy, acute kidney injury, and renal fibrosis, which are known to lead to renal dysfunction [15–20]. Interestingly, the G‐395A single‐nucleotide polymorphism (SNP) of the Klotho gene has been found to affect the expression of the sKl protein [15]. Despite the known associations of Klotho with both atrial fibrillation and renal function, at present, it is unclear whether the Klotho G‐395A polymorphism is also associated with the risk of atrial fibrillation in patients with MHD.

The present study aims to explore the correlation between atrial fibrillation and the Klotho G‐395A polymorphism in patients with MHD and elucidate the possible mechanisms affecting the occurrence of atrial fibrillation by also analyzing the differences in biochemical indicators between MHD patients with and without atrial fibrillation. The findings imply that this Klotho SNP could act as an early biomarker for the prevention and diagnosis of cardiovascular events in MHD patients.

## 2. Materials and Methods

### 2.1. Patient Selection

This study recruited 120 patients with MHD, including those with a normal sinus rhythm (*n* = 60), persistent atrial fibrillation (*n* = 30), and paroxysmal atrial fibrillation (*n* = 30), from Shanghai Fourth People’s Hospital between January 2022 and December 2023. Patients with a history of prior AF ablation, valvular heart surgery, or any cardiac surgery were excluded. Additionally, patients with known untreated thyroid dysfunction or abnormal TSH levels (> 4.5 or < 0.3 mIU/L) were excluded (*n* = 8). The following were the diagnostic criteria for atrial fibrillation [16] based on the ECG findings: irregular f wave instead of a P wave, frequency of about 350–600 times/min, irregular RR interval, and mostly normal QRS waveform. Paroxysmal atrial fibrillation is characterized by an AF waveform that automatically restores the sinus rhythm within 7 days, while persistent atrial fibrillation is characterized by an AF waveform that exceeds 7 days. The enrollment criteria were age ≥ 18 years, dialysis duration > 6 months, and occurrence of atrial fibrillation after dialysis. The exclusion criteria were age ≥ 80 years and presence of acute myocardial infarction, severe cardiac insufficiency, valvular heart disease, and congenital heart disease. A total of 120 healthy people with normal physical examination results were also selected from our hospital over the same period as the control group.

This study was approved by the Clinical Research Ethics Committee of Shanghai Fourth People’s Hospital (Approval No. 2022023‐001), and all the participants provided their signed informed consent.

### 2.2. Sample Collection and Analysis

Baseline data, including gender, age, dialysis age, and BMI (weight [kg]/height^2^[m^2^]), were obtained. Three fasting venous blood samples of 2 mL each were collected early morning on the day of dialysis. The first sample was immediately stored in a refrigerator with a temperature of −20°C till genotyping of the Klotho G‐395A gene was performed. The second sample was immediately centrifuged with temperature—25°C, rotation speed—3000 rpm, centrifugation time—15 min, and stored at −80°C. The blood samples was analyzed with the sKL ELISA kit (EHJ‐10235h; Jiangsu Huijia Medical Device Co. LTD, China) and iFGF23 ELISA kit (EHJ‐90801h; Jiangsu Huijia Medical Device Co. LTD, China) according to the manufacturers’ instructions. The final blood sample was assayed for biochemical indicators, such as calcium and phosphorus, using standard automated analyzer technology (Roche, Switzerland).

The diagnosis and classification of atrial fibrillation were confirmed based on the ECG results by an attending cardiologist at our hospital. The left atrial diameter was measured by a color ultrasound specialist using an echocardiograph (IE33; Philips, Netherlands) when the patient was in a relaxed state on the morning after dialysis. This timing was chosen to standardize volume conditions and reflect the “dry weight” state, minimizing confounding by fluid overload, consistent with previous studies [13, 14].

### 2.3. Detection of Klotho Gene Polymorphisms

A DNA separation kit (B518723; Shengong Bioengineering Co. LTD., China) was used to extract DNA from the whole‐blood samples according to the manufacturer’s instructions. TaqMan probe (3730XL; ABI, USA) was used to genotype the G‐395A locus of the Klotho gene. The G‐395A locus (rs1207568) was detected, and the nucleotide sequence was verified as CCCAAGTCGGG [G/A] AAAGTTGGTCG. The LightCycler 480 software was used to set up a fluorescence quantitative PCR instrument (Roche, Switzerland), and the samples were amplified and genotyped according to the fluorescence curve.

The following primers were used.

 Forward primer (RS1207568‐F): 5ʹ‐TTTCGTGGACGCTCAGGTT‐3ʹ
 Reverse primer (RS1207568‐R): 5ʹGTCCCAACGCAACCCATAA‐3ʹ

### 2.4. Statistical Analysis

SPSS22.0 was used to analyze the experimental data, and *p* < 0.05 was considered to indicate statistical significance. When the measurement data conformed to normal distribution, they were expressed as mean ± SD and were compared with a *t* test. For measurement data that did not conform to normal distribution, median and interquartile range were calculated and compared with the Mann–Whitney *U* test. Count data were expressed as the number of cases or percentages and were compared with the *X* ^2^ test. Pairwise correlations between experimental data were analyzed by Spearman correlation analysis. Binary logistic regression analysis was used to identify factors that were significant indicators of atrial fibrillation. For binary logistic regression, a stepwise forward selection method was used with entry criterion *p* < 0.05 and removal criterion *p* > 0.10. Collinearity was assessed, and variables with significant correlation (e.g., BNP and LA diameter: *r* = 0.48, *p* < 0.01; SBP and use of antihypertensive drugs) were handled through the stepwise procedure.

## 3. Results

### 3.1. Comparison of Genotypes Between the MHD Group and the Healthy Control Group

In our study, three genotypes—GG, GA, and AA—were detected. Due to the small sample size of genotype AA, the GA and AA genotypes were considered one group. The frequency of the GA/AA genotype and the A allele was significantly higher in the MHD patients (50.00% and 26.67%, respectively) than in the healthy control group (34.17% and 18.33%, respectively) (*p* < 0.05) (Table 1).

**TABLE 1:** Comparison of genotype distribution between MHD group and healthy group.

Groups	Number of cases	Genotype			Genotype		Allele genes	
		GG	GA	AA	GG %	GA/AA %	G %	A %
MHD group	120	60	56	4	60 50.00%	60 50.00%	88 73.33%	32 26.67%

Healthy group	120	79	38	3	79 65.83%	41 34.17%	98 81.67%	22 18.33%

*χ* ^2^		6.220			6.171		4.779	

*p*		0.043^∗^			0.013^∗^		0.029^∗^	

^∗^ *p* < 0.05.

### 3.2. Genotype Distribution Across the MHD Subgroups

The frequency of the GA/AA genotype and the A allele was significantly higher in MHD patients with atrial fibrillation than in MHD patients with normal sinus rhythm (*p* < 0.05). Furthermore, the GA/AA genotype and A allele frequencies were significantly higher in patients with persistent atrial fibrillation than in patients with paroxysmal atrial fibrillation (*p* < 0.05) (Table 2).

**TABLE 2:** Comparison of klotho genotypes and alleles among all groups in MHD patients.

Groups	Number of cases	Genotype			Allele genes		
		GG %	GA/AA %		G %	A %	
Sinus rhythm group	60	42 70.00%	18 30.00%	*χ* ^2^ = 25.496 *p* < 0.001^∗^	101 84.7%	19 15.84%	*χ* ^2^ = 14.403 *p* < 0.001^∗^
Atrial fibrillation group	60	18 30.00%	42 70.00%		75 62.50%	45 37.50%	

Paroxysmal atrial fibrillation group	30	13 43.33%	17 56.67%	*χ* ^2^ = 5.079 *p* ^=^0.024^*#*^	43 71.67%	17 28.33%	*χ* ^2^ = 4.302 *p* = 0.038^*#*^
Persistent atrial fibrillation group	30	5 16.67%	25 83.33%		32 53.33%	28 46.67%	

^∗^ *p* < 0.001 for the comparison between the sinus rhythm group and the atrial fibrillation group.

^#^ *p* < 0.05 for the comparison between the paroxysmal atrial fibrillation group and the persistent atrial fibrillation group.

### 3.3. Comparison of Clinical Parameters Between the MHD Subgroups

The serum sKl content in the atrial fibrillation groups was significantly lower than that in the sinus rhythm group, while serum iFGF23, atrial inner diameter, B‐type natriuretic peptide, and systolic blood pressure were significantly higher in the atrial fibrillation groups (*p* < 0.05) (Table 3). The serum sKl content was significantly lower, whereas the atrial inner diameter was significantly higher, in patients with persistent atrial fibrillation than in patients with paroxysmal atrial fibrillation (*p* < 0.05) (Table 4). No significant differences in serum potassium, magnesium, or TSH levels were observed between the AF and sinus rhythm groups, nor between persistent and paroxysmal AF groups (all *p* > 0.05). The most commonly used antiarrhythmics were beta‐blockers (metoprolol, bisoprolol) and amiodarone, with no significant differences between groups (*p* > 0.05) (Table 5).

**TABLE 3:** Comparison of clinical parameters between the atrial fibrillation group and sinus rhythm group in MHD.

	Atrial fibrillation group	Sinus rhythm group	*t*/*Z*/*χ* ^2^ value	*p* value
Age (years)	64.50 (53.00, 69.00)	55.50 (47.00, 68.00)	−1.624	0.105
Gender (male/female)	35/25	32/28	0.304	0.581
BMI (Kg/m^2^)	22.34 (20.02, 23.68)	22.56 (21.00, 25.20)	−1.646	0.100
SBP (mmHg)	146.00 (138.00, 150.00)	140.00 (125.75.147.50)	−2.217	0.027
DBP (mmHg)	83.50 (76.25, 88.75)	82.00 (78.25, 88.00)	−0.777	0.437
Dialysis age (m)	36.00 (12.00, 72.00)	33.00 (13.00, 72.00)	−0.292	0.770
Hb (g/L)	93.15 ± 18.03	98.75 ± 19.73	−1.623	0.107
Alb (g/L)	37.17 ± 6.70	37.67 ± 6.97	−0.399	0.691
Scr (umol/L)	1084.00 (984.50, 1207.00)	1183.00 (1016.00, 1314.75)	−1.924	0.054
BUN (mmol/L)	19.72 (15.38, 28.36)	20.31 (15.85, 24.41)	−0.619	0.536
UA (umol/L)	342.50 (271.50, 405.75)	385.00 (284.50, 476.75)	−1.459	0.144
Glu (mmol/L)	4.97 (4.45, 5.84)	4.83 (4.49, 5.85)	−0.430	0.667
TG (mmol/L)	1.29 (1.00, 1.71)	1.39 (0.94, 1.96)	−0.646	0.518
TC (mmol/L)	3.73 (2.93, 4.37)	3.64 (3.04, 4.20)	−0.701	0.483
Ca (mmol/L)	2.07 (1.95, 2.33)	2.19 (1.91, 2.38)	−0.648	0.517
Pi (mmol/L)	1.85 (1.28, 2.12)	1.81 (1.37, 2.36)	−0.837	0.402
iPTH (Pg/mL)	301.00 (193.23, 616.43)	279.25 (188.42, 520.50)	−0.714	0.475
Potassium (mmol/L)	4.21 (3.89, 4.58)	4.18 (3.92, 4.55)	−0.312	0.755
Magnesium (mmol/L)	0.89 (0.82, 0.97)	0.91 (0.84, 0.99)	−0.567	0.571
TSH (mIU/L)	1.89 (1.34, 2.56)	1.92 (1.41, 2.61)	−0.218	0.828
Vascular access (AVF/catheter)	48/12	50/10	0.340	0.560
BNP	8680.00 (6980.00, 11000.00)	7311.50 (4022.50, 9740.00)	−2.837	0.005
Left atrial diameter	42.00 (39.00, 47.00)	39.50 (37.00, 41.00)	−3.941	< 0.001
sKl (ng/mL)	16.93 (13.14, 22.64)	21.86 (16.92, 26.74)	−3.299	0.001
iFGF23 (ng/L)	464.56 (366.73, 567.25)	397.52 (347.68, 494.29)	−2.687	0.007

**TABLE 4:** Comparison of clinical parameters between persistent atrial fibrillation and paroxysmal atrial fibrillation.

	Paroxysmal atrial fibrillation group	Persistent atrial fibrillation group	*t*/*Z*/*χ* ^2^ value	*p* value
Age (years)	66.00 (55.00, 70.75)	62.00 (48.50, 68.25)	−1.798	0.072
Gender (male/female)	18/12	17/13	0.034	0.855
BMI (Kg/m^2^)	22.19 (20.22, 23.59)	22.68 (19.78, 23.73)	−0.274	0.784
SBP (mmHg)	143.50 (135.00, 150.00)	147.00 (140.00, 150.50)	−1.172	0.241
DBP (mmHg)	82.00 (76.00, 89.25)	84.50 (79.50, 88.25)	−0.163	0.871
Dialysis age (m)	24.00 (12.00, 72.00)	36.00 (12.00, 63.00)	−0.030	0.976
Hb (g/L)	93.93 ± 19.08	92.37 ± 17.20	0.334	0.740
Alb (g/L)	36.45 ± 5.73	37.90 ± 7.57	−0.834	0.408
Scr (umol/L)	1114.50 (981.75, 1285.00)	1048.00 (980.00, 1139.25)	−0.946	0.344
BUN (mmol/L)	19.20 (14.58, 26.94)	20.21 (15.83, 29.67)	−0.887	0.375
UA (umol/L)	336.50 (267.00, 390.00)	342.50 (277.50, 462.50)	−0.481	0.631
Glu (mmol/L)	4.91 (4.48, 5.85)	5.11 (4.34, 5.86)	−0.067	0.947
TG (mmol/L)	1.17 (0.85, 1.58)	1.30 (1.12, 1.98)	−1.619	0.105
TC (mmol/L)	3.74 (3.20, 4.31)	3.67 (2.59, 4.57)	−0.725	0.469
Ca (mmol/L)	2.14 (1.99, 2.32)	2.03 (1.89, 2.41)	−1.161	0.246
Pi (mmol/L)	1.94 (1.59, 2.10)	1.77 (1.19, 2.14)	−0.614	0.539
iPTH (Pg/mL)	301.50 (264.25, 690.60)	259.65 (157.15, 594.50)	−1.434	0.151
Potassium (mmol/L)	4.23 (3.91, 4.61)	4.19 (3.87, 4.55)	−0.401	0.689
Magnesium (mmol/L)	0.88 (0.81, 0.96)	0.90 (0.83, 0.98)	−0.478	0.633
TSH (mIU/L)	1.87 (1.32, 2.54)	1.91 (1.36, 2.58)	−0.215	0.830
BNP	8850 (7877.50, 15675.00)	8600 (6837.50, 11000.00)	−1.235	0.217
Left atrial diameter	40.00 (38.00, 43.00)	47.00 (41.00, 50.25)	−4.181	< 0.001
sKl (ng/mL)	19.66 (14.70, 24.40)	14.53 (12.31, 18.50)	−2.366	0.018
iFGF23 (ng/L)	421.97 (381.48, 509.91)	498.43 (327.40, 587.28)	−0.931	0.352

**TABLE 5:** Use of antiarrhythmic drugs in MHD patients.

Drug class	Sinus rhythm (*n* = 60)	Atrial fibrillation (*n* = 60)	Paroxysmal AF (*n* = 30)	Persistent AF (*n* = 30)	*p* value (AF vs. SR)	*p* value (parox vs. pers)
Beta‐blockers (metoprolol/bisoprolol)	28 (46.7%)	32 (53.3%)	15 (50.0%)	17 (56.7%)	0.467	0.599
Amiodarone	0 (0%)	18 (30.0%)	8 (26.7%)	10 (33.3%)	< 0.001	0.571
Other antiarrhythmics	2 (3.3%)	5 (8.3%)	2 (6.7%)	3 (10.0%)	0.423	0.662

There was no significant difference in the use of calcium or phosphorus binders between the sinus rhythm group and the atrial fibrillation group (*p* > 0.05) (Table 6).

**TABLE 6:** Comparison of the use of calcium–phosphorus binders in MHD patients among all groups.

Groups	Number of cases	Calcium and phosphorus modulating drugs		
		Phosphorus binder containing calcium	Phosphorus binding agent without calcium	
Sinus rhythm group	60	44	16	*χ* ^2^ = 0.409
Atrial fibrillation group	60	47	13	*p* = 0.522

The distribution of chronic kidney disease (CKD) etiologies (diabetic nephropathy, hypertensive nephrosclerosis, glomerulonephritis, polycystic kidney disease, etc.) did not differ significantly among the groups (*p* > 0.05) (Table 7). Regarding vascular access, the majority (*n* = 98, 81.7%) used arteriovenous fistula, while 22 patients (18.3%) used tunneled cuffed catheters, with no significant difference between AF and sinus rhythm groups (*p* = 0.34) (Table 3).

**TABLE 7:** Underlying etiologies of CKD in MHD patients.

Etiology	Sinus rhythm (*n* = 60)	Atrial fibrillation (*n* = 60)	Paroxysmal AF (*n* = 30)	Persistent AF (*n* = 30)	*p* value (AF vs. SR)	*p* value (parox vs. pers)
Diabetic nephropathy	24 (40.0%)	26 (43.3%)	13 (43.3%)	13 (43.3%)	0.713	1.000
Hypertensive nephrosclerosis	18 (30.0%)	19 (31.7%)	10 (33.3%)	9 (30.0%)	0.845	0.781
Glomerulonephritis	10 (16.7%)	9 (15.0%)	4 (13.3%)	5 (16.7%)	0.799	0.718
Polycystic kidney disease	4 (6.7%)	3 (5.0%)	2 (6.7%)	1 (3.3%)	0.695	0.557
Other/unknown	4 (6.7%)	3 (5.0%)	1 (3.3%)	2 (6.7%)	0.695	0.557

Although SBP and BNP were significant in univariate analysis, they were not retained in the final multivariate model due to stepwise selection, as they did not maintain independent significance after adjusting for LA diameter, GA/AA genotype, and iFGF23.

### 3.4. Correlation Analysis

**FIGURE 1:**
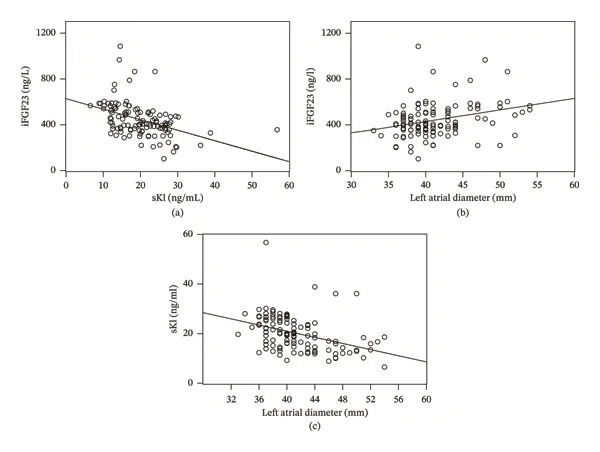
Correlation between sKl protein, iFGF23, and left atrial diameter.

Spearman correlation analysis showed that the serum sKl protein content was negatively correlated with the serum iFGF23 content (*r* = −0.514, *p* < 0.01) and left atrial diameter (*r* = −0.473, *p* < 0.01), and left atrial diameter was positively correlated with the serum iFGF23 level (*r* = 0.317, *p* < 0.01) (Figure 1).

### 3.5. Comparison of Clinical Parameters Between Different Genotypes

In MHD patients, the GA/AA genotype patients, the serum sKl level was significantly lower, and left atrial diameter was significantly higher, than that in patients with the GG genotype (*p* < 0.05) (Table 8).

**TABLE 8:** Comparison of clinical parameters between the GG genotype and GA/AA genotype in MHD.

	GG genotype	GA/AA genotype	*t*/*Z*/*χ* ^2^ value	*p* value
Age (years)	56.50 (51.00, 67.00)	64.50 (50.00, 70.00)	−1.292	0.196
Gender (male/female)	31/29	33/27	0.312	0.577
BMI (Kg/m^2^)	22.55 (21.46, 24.77)	22.52 (20.07, 23.73)	−1.299	0.194
SBP (mmHg)	140.00 (130.00, 148.00)	146.00 (136.50, 150.00)	−1.864	0.062
DBP (mmHg)	82.00 (78.00, 88.00)	82.00 (77.25, 88.00)	−0.129	0.897
Dialysis age (m)	29.00 (12.00, 72.00)	39.00 (12.00, 72.00)	−0.329	0.742
Hb (g/L)	97.48 ± 20.08	94.42 ± 17.96	0.8882	0.380
Alb (g/L)	37.05 ± 6.67	37.80 ± 6.99	−0.605	0.546
Scr (umol/L)	1166.00 (1003.00, 1306.50)	1085.50 (986.00, 1209.00)	−1.748	0.080
BUN (mmol/L)	22.12 (16.90, 28.38)	18.30 (14.33, 23.52)	−1.940	0.052
UA (umol/L)	365.50 (274.50, 452.00)	358.00 (286.00, 453.75)	−0.184	0.854
Glu (mmol/L)	4.91 (4.51, 6.00)	4.91 (4.48, 5.79)	−0.079	0.937
TG (mmol/L)	1.29 (0.94, 1.88)	1.30 (1.01, 1.92)	−0.021	0.983
TC (mmol/L)	3.74 (3.31, 4.24)	3.57 (2.68, 4.34)	−1.010	0.312
Ca (mmol/L)	2.14 (1.95, 2.33)	2.16 (1.95, 2.40)	−0.050	0.960
Pi (mmol/L)	1.80 (1.41, 2.34)	1.68 (1.22, 2.18)	−1.386	0.166
iPTH (Pg/mL)	281.56 (195.75, 575.55)	300.00 (186.65, 634.33)	−0.152	0.879
BNP (Pg/mL)	7931.00 (4655.00, 10415.00)	8517.00 (6905.00, 10825.00)	−1.192	0.233
Left atrial diameter (mm)	39.00 (37.00, 41.00)	41.00 (39.00, 46.00)	−3.564	< 0.001
sKl (ng/mL)	21.86 (16.17, 26.25)	17.23 (13.20, 23.39)	−2.393	0.017
iFGF23 (ng/L)	424.63 (362.72, 510.70)	410.78 (332.78, 543.70)	−0.323	0.747

### 3.6. Risk Factors for Atrial Fibrillation

Binary logistic regression showed that the GA/AA genotype, atrial inner diameter, and iFGF23 level are independent risk factors for atrial fibrillation in MHD patients. In particular, the GA/AA genotype carried a 5.444 times higher risk of atrial fibrillation than the GG genotype (Table 9).

**TABLE 9:** Logistic regression analysis of factors related to atrial fibrillation.

Independent variable	Β value	OR value	95% CI	*p* value
GA/AA	1.694	5.444	2.215–13.380	< 0.001
Left atrial diameter	0.128	1.137	1.018–1.269	0.022
iFGF23	0.005	1.005	1.001–1.009	0.011

## 4. Discussion

The present findings indicate that the allele A G‐395A polymorphism of the Klotho gene may act as a significant risk factor for the development of atrial fibrillation in patients undergoing MHD. The marker shows promise as an early biomarker, especially given the limitations in controlling other known risk factors such as hypertension and heart failure.

In this study, the distribution frequency of the G‐395A polymorphism at Site A of the Klotho gene was analyzed and found to be significantly higher in MHD patients than in the healthy controls. This observation supports previous reports that the Klotho G‐395A polymorphism may be a risk factor in patients with CKD. For example, Elghoroury et al. [17] found that the frequency of the polymorphic locus A gene carried by CKD children was significantly higher than in healthy children. Moreover, a Korean study showed that the incidence of end‐stage CKD and mortality was higher in carriers of the A gene than in noncarriers [18]. In animal experiments, it has been found that the Klotho gene can inhibit the development and progression of renal fibrosis by inhibiting the expression of Angiotensin II, fibroblast growth factor 2, transforming growth factor‐β1, and other such factors [21]. Correspondingly, the study found that decreased kidney function also affected the expression of the Klotho gene. Furthermore, a study of 236 patients with Stage 1–5 CKD found that the expression products of the Klotho gene were positively correlated with the patients’ glomerular filtration rate, and this conclusion remained valid even after adjusting for relevant factors such as age [22]. These findings together imply that the Klotho gene and its products are essential for kidney function and protection from pathogenic mechanisms, while the G‐395A polymorphism of this gene can adversely affect kidney function.

Mortality from cardiovascular disease in MHD patients is closely related to the occurrence of atrial fibrillation. Vazquez et al. [23] followed up a group of MHD patients for 4 years and found that the mortality rate in the atrial fibrillation group was three times that in the normal sinus rhythm group. The study further found that the incidence of atrial fibrillation increased with the progression of renal insufficiency, reaching more than 10% in patients with Stage 5 CKD [24]. Furthermore, in a prospective study of more than 40,000 patients with atrial fibrillation, after adjustment for relevant confounding factors such as age and blood sugar, the glomerular filtration rate was found to be negatively correlated with the incidence of atrial fibrillation, and renal failure was an independent risk factor for the occurrence of atrial fibrillation [25]. In this study, it was found that the carrier frequency of the A allele and distribution frequency of the A allele in MHD patients with atrial fibrillation were significantly higher than those in the normal sinus rhythm group (*p* < 0.05). Thus, the A allele may be related to the occurrence of atrial fibrillation in MHD patients. Accordingly, our clinical data showed that the left atrial diameter of GA/AA genotype patients was significantly greater than that of GG genotype patients (*p* < 0.05). As expectations, left atrial diameter emerged as a significant risk factor for atrial fibrillation in MHD patients according to logistic regression analysis. Importantly, the GA/AA genotype was identified as an independent risk factor in the binary logistic regression analysis. Surprisingly, systolic blood pressure and B‐type natriuretic peptide content, which are markers for hypertension and heart failure, respectively, known risk factors for the occurrence of atrial fibrillation, did not emerge as significant risk factors in our population. This points to the importance of the GA/AA genotype as a marker of atrial fibrillation in MHD patients.

The results of this study also demonstrate how mutations at the A allele site affect the activity of the Klotho protein. For example, the sKl protein content in carriers of the A gene was significantly lower than that in noncarriers. Further analysis of the relationship between the sKl protein and the occurrence of atrial fibrillation in MHD patients showed that the serum sKl content in the atrial fibrillation group was significantly lower than that in the normal sinus rhythm group, and the sKl content decreased with the persistence of atrial fibrillation. Thus, the decrease in serum sKl protein levels may be an important biochemical indicator of the occurrence and development of atrial fibrillation. In addition, the persistence of atrial fibrillation was positively correlated with the left atrial diameter, and this confirms the existence of atrial remodeling in patients with atrial fibrillation. This was corroborated by the results of the correlation analysis, which showed that the content of serum sKl protein was negatively correlated with left atrial diameter. Accordingly, animal studies have shown that mice with Klotho gene knockout had severe myocardial fibrosis and ventricular hypertrophy, while mice with Klotho gene overexpression had relatively mild myocardial fibrosis and ventricular hypertrophy [26]. Thus, the mechanism underlying the effect of the sKl protein on heart rhythm may be related to atrial remodeling caused by a decline in the levels of the sKl protein. Other studies have suggested that the arrhythmia caused by the decrease in sKl protein levels is related to the decrease in the number of ion channels on the surface of cardiomyocytes [27, 28]. The underlying mechanisms were not explored in detail in the present study and warrant further investigation in the future.

As iFGF23 from the FGF23 family is thought to bind to the FGFR1c/a‐Klotho complex and activate downstream signaling pathways [29], we also investigated the patterns of iFGF3 expression in our patient groups. The results showed that the serum iFGF23 level in the atrial fibrillation group was significantly higher than that in the sinus normal rhythm group, and correlation analysis indicated that the serum iFGF23 content was negatively correlated with the serum sKl content. Importantly, serum iFGF23 was identified as a risk factor for atrial fibrillation in MHD patients according to the results of logistic regression analysis. In line with our findings, Mathew et al. [30] found an association between serum iFGF23 and the incidence of atrial fibrillation in a 7‐ to 8‐year observational study of a population without underlying cardiovascular disease. In their study, iFGF23 contributed to the development of atrial fibrillation by promoting the formation of myocardial fibrosis. In contrast, Alonso et al. [31] observed no association between serum iFGF23 levels and the risk of atrial fibrillation in a large community cohort. Although our findings imply that serum iFGF23 might also be involved in the occurrence of atrial fibrillation, there was no significant difference in the serum iFGF23 content between the persistent atrial fibrillation group and the paroxysmal atrial fibrillation group. Based on the inconsistencies in these findings, further experimental studies are needed to investigate the role of iFGF23 in the development of atrial fibrillation.

Regarding vascular access, the distribution of arteriovenous fistulas and tunneled cuffed catheters was balanced between the atrial fibrillation and sinus rhythm groups (*p* = 0.34). Therefore, catheter‐related mechanical irritation of the right atrium is unlikely to explain the higher incidence of atrial fibrillation observed in our MHD patients.

According to the present results, Mizia‐Stec et al. [13] also reported that the occurrence of atrial fibrillation is associated with decrease in sKl and increase in iFGF23 in patients with end‐stage kidney disease. They also reported that the calcium and phosphorus levels changed with changes in sKl and iFGF23 and speculated that the occurrence of atrial fibrillation is related to the abnormal metabolism of calcium and phosphorus. In contrast to their observations, we did not observe any differences in serum calcium and blood phosphorus contents among MHD patients that were related to the occurrence of atrial fibrillation. This may reflect the compensatory regulation of calcium and phosphorus metabolism by serum sKl protein or serum iFGF23 in the early stage of cardiovascular disease [22]. In fact, Xie et al. [32] found in a study of patients with CKD that a decrease in serum sKl was an independent risk factor for myocardial damage independent of phosphorus metabolism. Thus, decreased serum sKl could be considered an early biomarker of atrial fibrillation in MHD patients, irrespective of the calcium and phosphorus contents. It should be noted, however, sKl did not emerge as an independent risk factor for atrial fibrillation according to logistic regression analysis in this study. This might be related to the small sample size of the study though.

Certain limitations of the present findings need to be considered when interpreting the results. First, the small sample size may have affected the results, and larger multicenter studies are needed to validate our findings. Furthermore, it is also necessary to exclude the influence of potential confounding factors, such as the number of hemodialysis sessions per week, the length of each session, and the adequacy of hemodialysis. Second, predialysis echocardiography was not routinely performed, limiting our ability to assess volume‐related changes in left atrial diameter. Future prospective studies should compare pre‐ and postdialysis measurements.

## 5. Conclusion

The occurrence of atrial fibrillation in MHD patients may be related to the existence of the Klotho G‐395A locus A allele. While serum sKl levels were reduced in patients with atrial fibrillation and showed a significant negative correlation with serum iFGF23 levels, further studies are required to verify their potential as biomarkers of atrial fibrillation in patients with MHD.

## Author Contributions

Guarantor of integrity of the entire study: Shunjie Chen.

Study concepts: Shunjie Chen.

Definition of intellectual content: Zhixiang Bian.

Literature research: Zhixiang Bian.

Clinical studies: Ying Zhang.

Data acquisition: Ying Zhang.

Data analysis: Zhixiang Bian.

Statistical analysis: Xiangxiang Wang.

Manuscript editing: XiaoXuan Su.

## Funding

This work was supported by the Research Startup Special Project of Shanghai Fourth People’s Hospital (sykyqd07901), the Key Discipline of Shanghai Health System (2024ZDXK0003), Clinical Key Specialty of Hongkou District (HKLCZD2024A01), the “Medicine + X” Cross‐disciplinary Research Project of Tongji University (2026‐0674‐ZD‐02), and the General Project of National Natural Science Foundation of China (82370693).

## Conflicts of Interest

The authors have no conflicts of interest.

## Data Availability

Data can be made available by the corresponding author on reasonable request.
